# Supplementary material for: Genetic diversity of whitefly species of the Bemisia tabaci Gennadius (Hemiptera: Aleyrodidae) species complex, associated with vegetable crops in Côte d’Ivoire
Source: PLoS One. 2022 Oct 31;17(10):e0276993. doi: 10.1371/journal.pone.0276993 (PMC9621431; doi:10.1371/journal.pone.0276993)
Supplement: S1 Table — (DOCX) [file pone.0276993.s001.docx]

**S1 Table.** Loci used for nuclear and mitochondrial DNA analysis

| **Primer name** | **References** | **Primer sequences** |
| --- | --- | --- |
| **Nuclear** | | |
| MS145 | (1) | F: CCTACCCATGAGAGCGGTAA |
|  |  | R: TCAACAAACGCGTTCTTCAC |
| P59 | (2) | F: CGGCGTTTCTCGTTTTCTT |
|  |  | R: TTTGCCAACTGAAGCACATCAATCA |
| P7 | (2) | F: AGGGTGTCAGGTCAGGTAGC |
|  |  | R: TTTGCGTAATAGAAAA |
| WF2H06 | (3) | F: TATTCGCCAATCGATTCCTT |
|  |  | R: CGGCGGAAATTTCGATAAA |
| WF1G03 | (3) | F: CTCCAAAATGGGACTTGAAC |
|  |  | R: GTAGAAGCCACACATACTAGCAC |
| WF1D04 | (3) | F: GTTGTTAGGTTACAGGGTTTGTC |
|  |  | R: GTCTTTACTTCTTTTCCTCCG |
| P5 | (2) | F: ATTAGCCTTGCTTGGGTCCT |
|  |  | R: TTTGCAAAAACAAAAGCATGTGTCAAA |
| CIRSSA2 | (4) | F: ACAATGCATGTTGATTGTGAA |
|  |  | R: TGAAAATGTCTACGGCCAGA |
| CIRSSA6 | (4) | F: CATATCGGTCATTATCCGCA |
|  |  | R: CATCAGGCTGGAAAGACGAG |
| CIRSSA7 | (4) | F: TGGCGATCCTCTTCTTGTTT |
|  |  | R: AAGAAGCAGCAGTTCATCCG |
| CIRSSA13 | (4) | F: AGTGCTGAAGGTCCACCGTA |
|  |  | R: GGGATTTCCAGGGGTTAAGA |
| CIRSSA41 | (4) | F: TGGGTGCATGGTTCTTACAG |
|  |  | R: TATCCGGTCGACAAACACAA |
| **Mitochondrial** |  |  |
| mtCOI | (5) | F: TGRTTTTTTGGTCATCCRGAAGT |
|  |  | R: TTTACTGCACTTTCTGCC |

**References used in this table :**

1. Dalmon A, Halkett F, Granier M, Delatte H, Peterschmitt M. Genetic structure of the invasive pest *Bemisia tabaci*: evidence of limited but persistent genetic differentiation in glasshouse populations. Heredity. 2008;100(3):316‑325.

2. Delatte H, David P, Granier M, Lett JM, Goldbach R, Peterschmitt M, et al. Microsatellites reveal extensive geographical, ecological and genetic contacts between invasive and indigenous whitefly biotypes in an insular environment. Genet Res. 2006;87(2):109‑124.

3. Hadjistylli M, Schwartz SA, Brown JK, Roderick GK. Isolation and characterization of nine microsatellite loci from *Bemisia tabaci* (Hemiptera: Aleyrodidae) Biotype B. J Insect Sci. 2014;14(1).

4. Ally HM. Genetic diversity and structure of the superabundant whitefly populations, vectors of viruses causing diseases of cassava in three East African countries (Malawi, Tanzania, and Uganda) [PhD Thesis]. La Réunion; 2019.

5. Mugerwa H, Seal S, Wang HL, Patel MV, Kabaalu R, Omongo CA, et al. African ancestry of New World, *Bemisia tabaci*-whitefly species. Sci Rep. 2018;8(1):1‑11.
