# Supplementary material for: Genetic diversity of whitefly species of the Bemisia tabaci Gennadius (Hemiptera: Aleyrodidae) species complex, associated with vegetable crops in Côte d’Ivoire
Source: PLoS One. 2022 Oct 31;17(10):e0276993. doi: 10.1371/journal.pone.0276993 (PMC9621431; doi:10.1371/journal.pone.0276993)
Supplement: S2 Table — (DOCX) [file pone.0276993.s002.docx]

**S2 Table.** All Bemisia tabaci mtCOI haplotypes and their accession numbers found in this study.

| **Locality** | **Plant species** | **Haplotype sequence name** | **N** | ***Bemisia tabaci* species** | **Accession Number** |
| --- | --- | --- | --- | --- | --- |
|  |  |  |  |  |  |
| **Bouaké** | Tomato | P5H7_CI_2019 | 2 | SSA3 | ON479263 |
| **Bouaké** | Tomato | P5E5_CI_2019 | 1 | SSA1_SG3 | ON479262 |
| **Ferkessédougou** | Tomato | P5D3_CI_2019 | 1 | MED Q1 | ON479245 |
| **Bouaké, Ferkessédougou, Korhogo** | Tomato, Eggplant, cucumber | P5G10_CI_2019 | 17 | MED Q1 | ON479246 |
| **Bouaké, Ferkessédougou, Korhogo** | Tomato, Eggplant, cucumber | P5G2_CI_2019 | 19 | MED Q1 | ON479244 |
| **Bouaké** | Tomato | P2E12_CI_2019 | 1 | MED Q1 | ON479243 |
| **Ferkessédougou, Korhogo** | Tomato, Cucumber | P4A1_CI_2019 | 8 | MED Q1 | ON479242 |
| **Bouaké** | Tomato | P5D7_CI_2019 | 1 | MED ASL | ON479247 |
| **Bondoukou, Agnibilékro, Abengourou, Bouaké, Bouaflé, Korhogo, Man** | Tomato, Eggplant, Cabbage, Green bean, Okra | P4E6_CI_2019 | 86 | MED ASL | ON479253 |
| **Bondoukou** | Tomato | P3D9_CI_2019 | 1 | MED ASL | ON479248 |
| **Bondoukou, Agnibilékro, Abengourou, Bouaké, Bouaflé** | Tomato, Eggplant, Cabbage | P1G3_CI_2019 | 32 | MED ASL | ON479250 |
| **Agnibilékro, Man** | Tomato, Eggplant, Okra | P2G5_CI_2019 | 1 | MED ASL | ON479249 |
| **Abengourou, Agnibilékro** | Tomato, Cabbage, Eggplant | P6C3_CI_2019 | 1 | MED ASL | ON479252 |
| **Bondoukou, Agnibilékro, Abengourou, Bouaké, Bouaflé, Ferkessédougou, Korhogo, Man, Odiénné** | Tomato, Eggplant | P3A11_CI_2019 | 11 | MED ASL | ON479251 |
| **Bondoukou** | Tomato | P3H11_CI_2019 | 1 | MED ASL | ON479255 |
| **Agnibilékro** | Tomato | P1C3_CI_2019 | 1 | MED ASL | ON479260 |
| **Korhogo** | Eggplant | P4B2_CI_2019 | 1 | MED ASL | ON479254 |
| **Abengourou, Agnibilékro, Bondoukou, Ferkessédougou, Odiénné** | Tomato, Eggplant, Cabbage, Cucumber | P4D12_CI_2019 | 9 | MED ASL | ON479257 |
| **Korhogo** | Eggplant | P4G2_CI_2019 | 1 | MED ASL | ON479256 |
| **Bondoukou, Agnibilékro, Abengourou, Bouaké, Bouaflé** | Tomato, Eggplant, Cabbage,cucumber, Green bean, Okra | P2A6_CI_2019 | 338 | MED ASL | ON479261 |
| **Agnibilékro** | Tomato | P2F8_CI_2019 | 1 | MED ASL | ON479258 |
| **Man** | Okra | P2C4_CI_2019 | 1 | MED ASL | ON479259 |
