# Supplementary material for: Genetic diversity of whitefly species of the Bemisia tabaci Gennadius (Hemiptera: Aleyrodidae) species complex, associated with vegetable crops in Côte d’Ivoire
Source: PLoS One. 2022 Oct 31;17(10):e0276993. doi: 10.1371/journal.pone.0276993 (PMC9621431; doi:10.1371/journal.pone.0276993)
Supplement: S3 Table — All bolded numbers are significant (Bonferroni corrected p-value). (DOCX) [file pone.0276993.s003.docx]

**S3 Table.** F_ST_ matrix of MED ASL populations. All bolded numbers are significant (Bonferroni corrected p-value).

| Sites | 1 | 2 | 3 | 4 | 5 | 6 | 7 | 8 | 9 | 10 | 11 | 12 | 13 |
| --- | --- | --- | --- | --- | --- | --- | --- | --- | --- | --- | --- | --- | --- |
| 1 | 0 |  |  |  |  |  |  |  |  |  |  |  |  |
| 2 | **0.0020** | 0 |  |  |  |  |  |  |  |  |  |  |  |
| 3 | **0.0371** | **0.0212** | 0 |  |  |  |  |  |  |  |  |  |  |
| 4 | **0.0381** | **0.0170** | **0.0102** | 0 |  |  |  |  |  |  |  |  |  |
| 5 | **0.0198** | **0.0271** | **0.0500** | **0.0376** | 0 |  |  |  |  |  |  |  |  |
| 6 | **0.0091** | **0.0049** | **0.0355** | **0.0342** | **0.0153** | 0 |  |  |  |  |  |  |  |
| 7 | **0.0172** | **0.0142** | **0.0582** | **0.0543** | **0.0299** | -0.0037 | 0 |  |  |  |  |  |  |
| 8 | **0.0078** | **0.0049** | **0.0363** | **0.0392** | **0.0215** | **-0.0016** | -0.0090 | 0 |  |  |  |  |  |
| 9 | **0.0363** | **0.0103** | **0.0302** | **0.0189** | **0.0681** | **0.0265** | **0.0372** | **0.0199** | 0 |  |  |  |  |
| 10 | **-0.0000** | **0.0104** | **0.0309** | **0.0331** | **0.0081** | **0.0112** | **0.0242** | **0.0143** | **0.0476** | 0 |  |  |  |
| 11 | -0.0021 | 0.0008 | **0.0276** | **0.0326** | **0.0235** | **0.0077** | **0.0333** | **0.0162** | **0.0352** | **0.0035** | 0 |  |  |
| 12 | **0.0352** | **0.0096** | **0.0080** | **0.0100** | **0.0462** | **0.0158** | **0.0284** | **0.0189** | **0.0082** | **0.0348** | **0.0284** | 0 |  |
| 13 | **0.0070** | 0.0010 | **0.0102** | **0.0121** | **0.0246** | **0.0098** | **0.0281** | **0.0094** | **0.0166** | **0.0065** | **0.0040** | **0.0102** | 0 |
